# Glances at the Hospitals

**Published:** 1900-02-03

**Authors:** 


### GLANCES AT THE HOSPITALS.

### THE KILMARNOCK INFIRMARY.

This hospital was opened in 1868, and it now issues ts thirty-first annual report dealing with the statistics for the year ending November 11th, 1899. The institution then contained 111 patients. Of these 26 were medical cases, 29 surgical, and 56 fever. There were 1,103 admissions during the year, divisible into 344 fever cases, 338 medical, and 421 surgical. An analysis of these shows that 212 cases of scarlet fever were treated, with the result that 201 recovered and 11 died; of 91 cases of enteric fever 13 died; of 16 cases of diphtheria 2 died; and of 7 cases of erysipelas 1 died. Of the 428 surgical cases treated to a termination we find that 286 were cured, 77 improved, and 43 died, the remainder being dismissed or not improved. Of the 336 medical cases treated to a termination 174 recovered, 82 were improved, and 50 died; the remaining 30 were not improved or were dismissed. When compared with the statistics of 1898 the numbers show that 89 more patients were treated in 1899 than in the previous year; and, as the directors point out, this increase in numbers must mean an increase of expenditure under various heads. In addition to this increase of expenditure caused by the number of patients under treatment it has been found necessary to increase the accommodation for the nursing staff, and a sum of £1,200 is now being spent on a building for that purpose. The total income for the year was £4,872, and obtained as follows: £801 in general subscriptions; workpeople's contributions, £791; and £232 in Congregational collections. The balance was derived from interest on money invested. Under some of these headings there was a decrease on the previous year, but we are glad to be able to record that the workpeople's own subscriptions increased by £60, and we trust this may be accepted as an indication of two important features: satisfactory state of the labour market and a laudable interest of the workpeople in the welfare of the hospital. The honorary directorate of the infirmary includes the Duke of Portland, the Earl of Eglinton, and the Earl of Glasgow. Mr. Arbuckle is president, and the medical and surgical staffs are represented by Dr. McAlister, Dr. Frew, Dr. Robertson, Dr. Lawrie, Dr. Macleod, Dr. W. McAlister, and Dr. Prentice. Mr. Lipscomb is dental surgeon, and Mr. Austin is secretary.

